# A New Phloroglucinol Diglycoside Derivative from *Hypericum japonicum* Thunb.

**DOI:** 10.3390/molecules13112796

**Published:** 2008-11-07

**Authors:** Xiao Wei Wang, Yu Mao, Nai-Li Wang, Xin Sheng Yao

**Affiliations:** 1 Department of Natural Products Chemistry, Shenyang Pharmaceutical University, 103. Wenhua Road Shenhe District, Shenyang, Liaoning 110016, P.R. China; E-mail: wangxiaowei@szda.gov.cn (X-W. W.); 2 Shenzhen Institute for Drug Control, Shenzhen, Guangdong 518029, P.R. China; E-mail: maoyu_007@163.com (Y. M.)

**Keywords:** *Hypericum japonicum* Thunb, 4,6-dimethyl-1-*O*-[α-L-rhamnopyranosyl-(1→6)-β-D-glucopyranosyl] multifidol, Antihypoxic activity

## Abstract

A new phloroglucinol diglycoside **1**, together with eight known compounds, were isolated from *Hypericum japonicum* Thunb*.* The structure of the new compound **1** was determined by spectroscopic methods to be 4,6-dimethyl-1-*O*-[α-L-rhamnopyranosyl-(1→6)-β-D-glucopyranosyl] multifidol. Different solvent extracts of *Hypericum japonicum* Thunb*.* were tested for *in vivo* antihypoxic activity using mice, with the EtOAc extract showing better activity.

## Introduction

**Figure 1:**
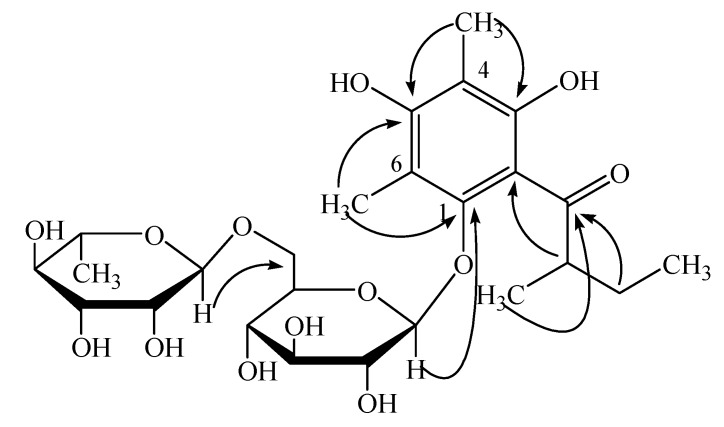
Key HMBC correlations of compound **1 **(^1^H →^13^C).

*Hypericum japonicum* Thunb. is an annual herb from the genus *Hypericum* L. (Clusiaceae/ Hypericaceae). The whole plant has been used for the treatment of several bacterial diseases, infectious hepatitis, gastrointestinal disorder and tumors [1]. As part of our search for antihypoxic ingredients from Chinese herbs, we carried out an activity screening study, during which we found that a 60% EtOH extract of *Hypericum japonicum* Thunb. showed better antihypoxic activity than other herb extracts. In continuation of this research, a new glycoside **1** and eight known compounds **2-9** (Figure 1) have been isolated by silica gel and ODS column chromatography and preparative HPLC. This paper deals with the isolation of these nine constituents, the structure elucidation of the new glycoside, and their antihypoxic activities.

## Results and Discussion

Compound **1** (Figure 1) was obtained as brown amorphous powder, 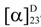-24.5°(*c*. 0.5, MeOH), m.p. 220～223°C. Its molecular formula was deduced as C_25_H_38_O_13_ from the quasi-molecular ion peak at 569.2200 ([M + Na]^+^, calcd. 569.2260) in HR-ESI-MS spectrum. The IR spectrum exhibited the absorptions at 3350 cm^-1^ (OH), 1630 cm^-1^ (carbonyl), 1598 cm^-1^ and 1586 cm^-1^ (phenyl). Its ^1^H-NMR spectrum (Table 1) showed one hydroxyl proton at *δ* 12.48 (1H, br s). It also showed five methyl groups at *δ* 2.10 (3H, s), 1.97 (3H, s), 1.07 (3H, d, *J* = 6.1 Hz), 0.91 (3H, d, *J* = 7.2 Hz) and 0.87 (3H, t, *J* =7.5 Hz), and two anomeric protons at *δ* 4.42 (1H, br s) and 4.29 (1H, d, *J* = 7.7 Hz). The ^13^C-NMR spectrum showed six aromatic signals (*δ* 160.0, 158.3, 152.7, 110.1, 109.9, 107.5) and one carbonyl signal (*δ* 210.0). Combining with DEPT and ^1^H-NMR data, we deduced that the benzene ring was completely substituted. Signals at *δ* 3.0～3.5 in the ^1^H-NMR spectrum and signals at *δ* 60～80 in the ^13^C-NMR spectrum suggested two sugar units.

Compound **1** was hydrolyzed with 2 M HCl/CH_3_OH solution to give 4,6-dimethylmultifidol, 4,6-dimethylmultifidol glucoside, one disaccharide and two monosaccharides. Multifidol and multifidol glucoside had been previously identified in the latex of *Jatropha multifida* in 1989 [2].. Two phloroglucinol glycosides: 2,6-dihydroxy-3,5-dimethyl-1-isobutyrylbenzene-4-*O*-β-D-glucoside and 2,6-dihydroxy-3,5-dimethyl-1-(2-methylbutyryl)benzene-4-*O*-β-D-glucoside had been previously identified in *Hypericum japonicum* [3]. These were very similar to “multifidol glucoside” in chemical structure, except for the glucose linkage with multifidol. The two monosaccharides were identified as rhamnose and glucose by GC-MS analysis using authentic monosaccharide samples as references. Combing the *J* value between the anomeric proton and the 2-H proton in each monosaccharide with the optical rotation values (the 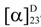 of rhamnose is -4.4°, the 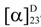 of glucose is +52.5°), the configuration of the rhamnose was identified as α-L, while that of the glucose was β-D. Thus, compound **1** was identified as 4,6-dimethyl-1-*O*-[α-L-rhamnopyranosyl-(1→6)-β-D-glucopyranosyl] multifidol. The complete assignments of ^1^H- and ^13^C-NMR data were based on the analyses of HSQC, ^1^H-^1^H COSY, and HMBC spectra (Figure 1). The key ^1^H-^13^C long-range correlations could be observed from H-1 of the rhamnose unit at *δ* 4.42 to C-6 of the glucopyranose unit at *δ* 67.4, from H-1 of the glucopyranose unit at *δ* 4.29 to C-1 of the phenyl unit at *ä* 152.7, and from H-2 of the methylbutyryl chain at *δ* 3.88 to C-2 of the phenyl unit at *δ* 110.1 in the HMBC spectrum.

**Table 1:** ^1^H-NMR and ^13^C-NMR (DMSO-*d*_6 _) data of compound **1**.

Position	δ_H_	δ_C_	Position	δ_H_	δ_C_
Methylbutyryl chain			Glucopyranose unit		
1	-	210.0	1	4.29 (1H, d, 7.7)	104.0
2	3.88 (1H, m)	44.9	2	3.28 (1H, m)	74.1
2-CH_3_	0.91 (3H, d, 7.2)	17.9	3	3.19 (1H, m)	76.2
3	1.31 (1H, m) 1.80 (1H, m)	24.6	4	3.30 (1H, m)	70.7
4	0.87 (3H, t, 7.5)	11.9	5	3.10 (1H, m)	75.0
Phenyl unit			6	3.69 (2H, d, 10.5)	67.4
1	-	152.7	Rhamnose unit		
2	-	110.1	1	4.42 (1H, br s)	101.0
3	-	158.3	2	3.50 (1H, m)	70.1
4	-	107.5	3	3.29 (1H, m)	70.2
4-CH_3_	1.97 (3H, s)	8.6	4	3.13 (1H, m)	72.0
5	-	160.0	5	3.27 (1H, m)	68.3
6	-	109.9	6	1.07 (3H, d, 6.1)	17.8
6-CH_3_	2.10 (3H, s)	9.5			

**Figure 2:**
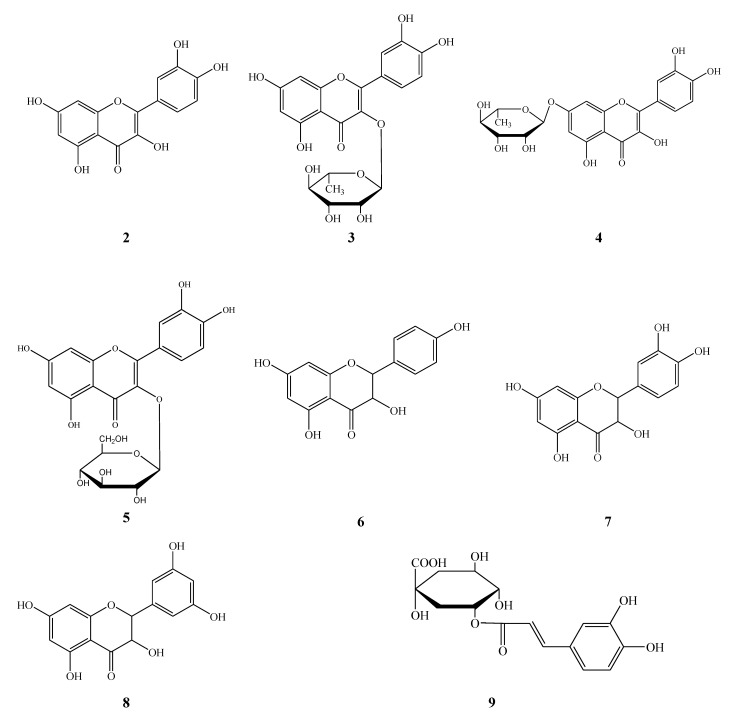
The structures of compounds **2-9** isolated from *Hypericum japonicum* Thunb.

The other eight known compounds were identified as quercetin (**2**), quercitrin (quercetin 3-*O*-L-rhamnoside) (**3**), quercetin 7-*O*-L-rhamnoside (**4**), isoquercitrin (quercetin 3-*O-β-*D-glucoside) (**5**), dihydrokaempferol (**6**), dihydroquercetin (**7**), 3,5,7,3’,5'-pentahydroxydihydroflavonol (**8**) and chlorogenic acid (**9**) by direct comparisons of their NMR data with literature data. The structures of compounds **2**-**9** are shown in Figure 2.

### Antihypoxic activity of Hypericum japonicum *Thunb* extracts

Male mice (body weight: 18±2g) were randomly divided into a test group and a control group.. Before the mice were placed under hypoxic conditions for 2 h, the test substances (CHCl_3_ extract, EtOAc extract, *n*-BuOH extract and water extract, 20 mg/0.5 mL, equivalent to 1g/kg body weight) and the control solution (DMSO diluted solution, 0.5 mL) were administrated to mice by gastric perfusion. Then each mouse was placed in a 150 mL flask and the flasks were sealed with rubber plugs. Under the sealed and hypoxic situation, the tolerance time in both groups were recorded. The results were as follows: 

**Table 2:** The tolerance time of mice administrated different extracts of *Hypericum japonicum* Thunb.

Substances	Dose	Mice number (n)	Tolerance time (min)
DMSO diluted solution	0.5 mL	20	33.30 ± 7.23
60% EtOH extract	20 mg/0.5 mL	10	38.47 ± 9.22
CHCl_3_ extract	20 mg/0.5 mL	10	40.15 ± 9.58
EtOAc extract	20 mg/0.5 mL	10	**44.37 ± 10.25**
*n*-BuOH extract	20 mg/0.5 mL	10	38.37± 8.18
Water extract	20 mg/0.5 mL	10	33.62 ± 11.30

## Conclusions

From the study, the EtOAC extract of *Hypericum japonicum* Thunb. was proven to have antihypoxic activity. A new phloroglucinol diglycoside, 4,6-dimethyl-1-*O*-[α-L-rhamnopyranosyl-(1°6)-β-D-gluco-pyranosyl] multifidol, along with eight known compounds, were separated from the active fraction.

## Experimental

### General

Optical rotations were determined on a JASCO P-1020 polarimeter in MeOH and HR-ESI-MS spectra were obtained on a Micromass Q-TOF mass spectrometer. NMR spectra were recorded on a Bruker AVANCE 400 NMR spectrometer (400 MHz for ^1^H, 100 MHz for ^13^C). The NMR data were measured in DMSO-*d*_6_ with tetramethylsilane (TMS) as internal standard. IR spectra were recorded on a FTIR 8400 spectrophotometer (Shimadzu, Japan) using KBr discs as stated. UV spectra were recorded on a UV2401PC spectrophotometer (Shimadzu, Japan). Column chromatography was carried out on silica gel H60 (Qingdao Haiyang Chemical Group Corporation, Qingdao, P.R. China), Sephadex LH-20 (Amersham Biosciences AB) and ODS (60~80 μm, Merck) as packing materials.

### Plant material

In our experiments we used the whole plant. *Hypericum japonicum* Thunb. herbs were collected from GuangXi province in P.R. China, in July 2003 and identified by Prof. Weichun Wu (Department of Medical Plants, Shenyang Pharmaceutical University, P.R. China). A voucher specimen was deposited at the Department of Natural Products Chemistry of Shenyang Pharmaceutical University.

### Extraction

The 60% EtOH extract of *Hypericum japonicum* Thunb. (4.3 kg) was dissolved and suspended in water and partitioned between chloroform, ethyl acetate and *n*-BuOH, respectively (three times each). Four fractions, that is, a chloroform fraction (83 g), an ethyl acetate fraction (114 g), an *n*-butanol fraction (153 g) and an aqueous fraction (228 g) were obtained after evaporation of the corresponding solvents *in vacuo*. The EtOAc fraction was subjected to silica gel column chromatography (SiO_2_, 800 g, eluted with 100:0→50:50 CHCl_3_/MeOH) to obtain fractions E1-E14.. Fraction E2 fraction (2.6 g) was applied to a Sephadex LH-20 column and eluted with CHCl_3_/MeOH (1:1) to give a subfraction which was subjected to ODS column chromatography with eluted with a H_2_O/MeOH gradient with increasing MeOH percent, to yield **1** (5.5 mg), **2** (15.3 mg), **3** (20.6 mg) , **4** (19.4 mg) and **5** (10.7 mg). Fraction E14 (2.3 g) was chromatographed over Sephadex LH-20 column (CHCl_3_-MeOH=1:1) and purified using preparative HPLC (ODS, 35%～60% MeOH) to yield **6** (6.4 mg), **7** (8.1 mg) and **8 **(5.9 mg). Fraction E12 (3.4 g) was chromatographed over Sephadex LH-20 column (CHCl_3_-MeOH=1:1) to yield **9** (1.3 g). Their structures are all shown in Figure 2..

*Quercetin* (**2**) [4]: C_15_H_10_O_7_; yellow powder (MeOH); HCl-Mg reaction (+); ESI-MS (positive) *m/z* 301 [M-H]^+^; UV (MeOH) λ_max_ nm: 369, 258, 208; IR *ν*_max_ (KBr): 3450 (br, OH), 2950, l670, l630, l550, l510, 1310 cm^-1^; ^1^H-NMR *δ*: 12.49 (1H, s, OH-5), 10.78 (1H, s, OH-7), 9.59 (1H, s, OH-4’), 9.36 (1H, s, OH-3), 9.30 (1H, s, OH-3’), 7.67 (1H, d, *J* = 2.2 Hz, H-2’), 7.53 (1H, dd, *J* = 8.5, 2.2 Hz, H-6’), 6.88 (1H, d, *J* = 8.5 Hz, H-5’), 6.41 (1H, d, *J* = 2.0 Hz, H-8), 6.18 (1H, d, *J* = 2.0 Hz, H-6); ^13^C-NMR *δ*: 175.8 (C-4), 163.9 (C-7), 160.7 (C-9), 156.1 (C-5), 147.7 (C-4’), 146.8 (C-2), 145.0 (C-3’), 135.7 (C-3), 121.9 (C-1’), 119.9 (C-6’), 115.6 (C-5’), 115.0 (C-2’), 103.0 (C-10), 98.2 (C-6), 93.3 (C-8).

*Quercitrin (quercetin 3-O-L-rhamnoside)* (**3**) [5]: C_21_H_20_O_11_; yellow powder (MeOH); HCl-Mg reaction (+); Molish reaction (+); ESI-MS (positive) *m/z* 471 [M+Na]^+^, 447[M-H]^+^; UV (MeOH) λ_max_ nm: 355, 265, 257; ^1^H-NMR *δ*: 12.66 (1H, s, OH-5), 11.00 (1H. s, OH-7), 9.83 (1H. s, OH-4’), 9.42 (1H. s, OH-3’), 7.31 (1H, d, *J* = 2.0 Hz, H-2’)7.27 (1H, dd, *J* = 8.3, 2.0 Hz, H-6’), 6.88 (1H, d, *J* = 8.3 Hz, H-5’), 6.41 (1H, d, *J* = 2.0 Hz, H-8), 6.22 (1H, d, *J* = 2.0 Hz, H-6), 5.27 (1H, br s, rha-H-1), 3.99 (1H, m, rha-H-2), 3.55 (1H, m, rha-H-3), 3.18 (1H, m, rha-H-5), 3.10 (1H, m, rha-H-4), 0.82 (3H, d, *J* = 5.9 Hz, rha-H-5-CH_3_); ^13^C-NMR *δ*: 177.9 (C-4), 164.3 (C-7), 161.4 (C-5), 157.5 (C-9), 156.6 (C-2), 148.6 (C-4’), 145.3 (C-3’), 134.4 (C-3), 121.3 (C-6’), 120.9 (C-1’), 115.8 (C-5’), 115.6 (C-2’), 104.2 (C-10), 101.9 (rha-C-1), 98.8 (C-6), 93.8 (C-8), 71.3 (rha-C-4), 70.7 (rha-C-3), 70.5 (rha-C-2), 70.2 (rha-C-5), 17.6 (rha-C-5-CH_3_).

*Quercetin 7-O-L-rhamnoside* (**4**) [6]: C_21_H_20_O_11_; yellow powder (MeOH); HCl-Mg reaction (+); Molish reaction (+); ESI-MS (positive) *m/z* 471 [M+Na]^+^, 447 [M-H]^+^; ^1^H-NMR *δ*: 12.49 (1H, s, OH-5), 9.48 (1H, s, OH-4’), 7.73 (1H, d, *J* = 2.2 Hz, H-2’), 7.59 (1H, dd, *J* = 8.5, 2.2 Hz, H-6’), 6.90 (1H, d, *J* = 8.5 Hz, H-5’), 6.79 (1H, d, *J* = 2.1 Hz, H-8), 6.42 (1H, d, *J* = 2.1 Hz, H-6), 5.55 (1H, d, *J* = 1.4 Hz, rha-H-1), 3.86 (1H, m, rha-H-2), 3.66 (1H, m, rha-H-3), 3.46 (1H, m, rha-H-5), 3.32 (1H, m, rha-H-4), 1.14 (3H, d, *J* = 6.1 Hz, rha-H-5-CH_3_); ^13^C-NMR *δ*: 175.9 (C-4), 161.4 (C-7), 160.3 (C-5), 155.7 (C-9), 147.9 (C-2), 147.5 (C-4’), 145.1 (C-3’), 136.1 (C-3), 121.8 (C-1’), 120.1 (C-6’), 115.6 (C-2’), 115.2 (C-5’), 104.6 (C-10), 98.8 (rha-C-1), 98.4 (C-6), 94.2 (C-8), 71.6 (rha-C-4), 70.3 (rha-C-3), 70.1 (rha-C-2), 69.8 (rha-C-5), 17.9 (rha-C-5-CH_3_).

*Isoquercitrin(quercetin 3-O-β-D-glucoside)* (**5**) [7]: C_21_H_20_O_12_; yellow powder (MeOH); HCl-Mg reaction (+); Molish reaction (+); ESI-MS (positive) *m/z* 487 [M+Na]^+^, 463 [M-H]^+^; IR *ν*_max_ (KBr): 3375, 1660, 1606, 1494, 1362, 1304, 1200, 1061, 801, 595 cm^-1^; ^1^H-NMR *δ*: 12.64 (1H, s, OH-5), 10.78 (1H, s, OH-7), 9.60 (1H, s, OH-4’), 9.16 (1H, s, OH-3’), 7.58 (1H, dd, *J* = 9.0, 2.2 Hz, H-6’), 7.56 (1H, d, *J* = 2.2 Hz, H-2’), 6.84 (1H, d, *J* = 9.0 Hz, H-5’), 6.40 (1H, d, *J* = 2.1 Hz, H-8), 6.20 (1H, d, *J* = 2.0 Hz, H-6), 5.46 (1H,d, *J* = 7.5 Hz, glu-H-1), 4.00～3.20 (4H, m, glu-H-2～5), 3.57 (2H, d, *J* = 11.4 Hz, glu-H-6); ^13^C-NMR *δ*: 177.4 (C-4), 164.1 (C-7), 161.2 (C-5), 156.3 (C-2), 156.2 (C-9), 144.8 (C-3’), 148.4 (C-4’), 133.3 (C-3), 121.6 (C-6’), 121.1 (C-1’), 116.2 (C-5’), 115.2 (C-2’), 104.0 (C-10), 100.9 (glu-C-1), 98.6 (C-6), 93.5 (C-8), 77.5 (glu-C-3), 76.5 (glu-C-5), 74.1 (glu-C-2), 69.9 (glu-C-4), 61.0 (glu-C-6).

*Dihydrokaempferol* (**6**) [8]: C_15_H_12_O_6_; colorless needle (MeOH); HCl-Mg reaction (+); Molish reaction (-); ESI-MS (positive) *m/z* 311 [M+Na]^+^, 287 [M-H]^+^; ^1^H-NMR *δ*: 11.90 (1H, s, OH-5), 10.84 (1H, s, OH-7), 9.55 (1H, s, OH-4’), 7.30 (2H, d, *J* = 8.4 Hz, H-2’, 6’), 6.78 (2H, d, *J* = 8.4 Hz, H-3’, 5’), 5.91 (1H, d, *J* = 2.0 Hz, H-8), 5.86 (1H, d, *J* = 2.0 Hz, H-6), 5.75 (1H, br s, OH-3), 5.05 (1H, d, *J* = 11.4 Hz, H-2), 4.58 (1H, d, *J* = 11.4 Hz, H-3); ^13^C-NMR *δ*: 197.8 (C-4), 166.8 (C-7), 163.3 (C-5), 162.5 (C-9), 157.7 (C-4’), 129.4 (C-1’), 127.5 (C-2’, 6’), 114.9 (C-3’, 5’), 100.4 (C-10), 96.0 (C-6), 95.0 (C-8), 82.9 (C-2), 71.4 (C-3).

*Dihydroquercetin* (**7**) [9]: C_15_H_12_O_7_; white needle (MeOH); HCl-Mg reaction (+); Molish reaction (-); ESI-MS (positive) *m/z* 303 [M-H]^+^; UV (MeOH) λ_max_ nm: 327, 289; IR *ν*_max_ (KBr): 3410 (OH); 1645 (C=O); 1610, l505 (phenyl C=C); 1120, 975, 770; ^1^H-NMR *δ*: 11.89 (1H, s, OH-5), 10.81 (1H, s, OH-7), 9.01 (1H, s, OH-4’), 8.96 (1H, s, OH-3’), 6.87 (1H, s, H-6’), 6.74 (2H, s, H-2’, 5’), 5.90 (1H, d, *J* = 2.1 Hz, H-8), 5.85 (1H, d, *J* = 2.1 Hz, H-6), 4.98 (1H, d, *J* = 11.1 Hz, H-2), 4.50 (1H, d, *J* = 11.1 Hz, H-3); ^13^C-NMR *δ*: 197.7 (C-4), 166.7 (C-7), 163.3 (C-5), 162.5 (C-9), 145.7 (C-4’), 144.9 (C-3’), 128.0 (C-1’), 119.3 (C-6’), 115.3 (C-2’), 115.1 (C-5’), 100.5 (C-10), 95.9 (C-6), 94.9 (C-8), 83.0 (C-2), 71.5 (C-3).

*3,5,7,3’,5'-Pentahydroxydihydroflavonol* (**8**) [10]: C_15_H_12_O_7_; yellow powder (MeOH); HCl-Mg reaction (+); Molish reaction (-); ESI-MS (positive) *m/z* 303 [M-H]^+^; ^1^H-NMR *δ*: 11.89 (1H, s, OH-5), 10.86 (1H, s, OH-7), 8.93 (1H, s, OH-5’), 8.92 (1H, s, OH-3’), 6.93 (1H, d, *J* = 1.7 Hz, H-4’), 6.72 (2H, d, *J* = 1.7 Hz, H-2’, 6’), 6.18 (1H, d, *J* = 6.2 Hz, H-2), 5.92 (1H, d, *J* = 2.1 Hz, H-8), 5.91 (1H, d, *J* = 2.1 Hz, H-6), 5.33 (1H, d, *J* = 2.4 Hz, OH-3), 4.02 (1H, dd, *J* = 6.2, 2.4 Hz, H-3). ^13^C-NMR *δ*: 195.5 (C-4), 166.7 (C-7), 163.9 (C-5), 162.7 (C-9), 144.7 (C-5’), 145.2 (C-3’), 127.0 (C-1’), 118.6 (C-6’), 115.3 (C-2’), 114.9 (C-4’), 100.2 (C-10), 95.8 (C-6), 94.9 (C-8), 81.0 (C-2), 70.9 (C-3).

*Chlorogenic acid* (**9**) [11]: C_16_H_18_O_9_; light yellow powder (MeOH); HCl-Mg reaction (-); FeCl_3_ reaction (+);ESI-MS (positive) *m/z* 377 [M+Na]^+^, 353[M-H]^+^; UV (MeOH) λ_max_ nm: 204.5 sh, 218, 246, 296..5 sh, 329.5; IR *ν*_max_ (KBr): 3412 (br), 2928, l689, l628, l604, 15l8, 1374, l275, l181, 1160，1120, 1082，1040, 977, 853, 813; ^1^H-NMR *δ*: 9.55 (1H, s, caf-OH-7), 9.18 (1H, s, caf-OH-6), 7.46 (1H, d, *J* = 15.9 Hz, caf-H-3), 7.02 (1H, d, *J* = 2.0 Hz, caf-H-5), 6.95 (1H, dd, *J* = 8.2, 2.0 Hz, caf-H-9), 6.76 (1H, d, *J* = 8.2 Hz, caf-H-8), 6.19 (1H, d, *J* = 15.9 Hz, caf-H-2), 5.16 (1H, m, qui-H-3), 3.86 (1H, m, qui-H-5), 3.63 (1H, m, qui-H-4), 1.88 (2H, m, qui-H-2), 1.99 (2H, m, qui-H-6). ^13^C-NMR *δ*: 176.0 (qui-C-1-COOH), 166.1 (caf-C-1), 148.1 (caf-C-7), 145.6 (caf-C-6), 144.4 (caf-C-3), 125.7 (caf-C-4), 121.1 (caf-C-9), 115.8 (caf-C-5), 115.0 (caf-C-2), 114.6 (caf-C-8), 73.0 (qui-C-1), 71.3 (qui-C-4), 70.4 (qui-C-3), 67.7 (qui-C-5), 35.1 (qui-C-2), 34.9 (qui-C-6).
